# KRS-Net: A Classification Approach Based on Deep Learning for Koi with High Similarity

**DOI:** 10.3390/biology11121727

**Published:** 2022-11-29

**Authors:** Youliang Zheng, Limiao Deng, Qi Lin, Wenkai Xu, Feng Wang, Juan Li

**Affiliations:** 1 College of Mechanical and Electrical Engineering, Qingdao Agricultural University, Qingdao 266109, China; 2 College of Science and Information Science, Qingdao Agricultural University, Qingdao 266109, China; 3 Key Laboratory of Cultivation and High-Value Utilization of Marine Organisms in Fujian Province, Xiamen 361013, China; 4 College of Marine Science and Engineering, Qingdao Agricultural University, Qingdao 266109, China

**Keywords:** deep learning, classification, AI (artificial intelligence), object recognition, fish

## Abstract

**Simple Summary:**

The diversity of fish resources is an important component of biodiversity. As a branch of fish, the diversity of koi varieties is conducive to improving the genetic quality of offspring, avoiding inbreeding, and improving their adaptability to the natural environment. The variety classification of koi is a necessary step to improve the diversity of koi varieties and breeding quality. The traditional manual classification method of koi variety faces some problems, such as high subjectivity, low efficiency, and high misclassification rate. Therefore, we studied an intelligent method of classifying koi variety using an artificial intelligence approach, and designed a deep learning network model, KRS-Net. The intelligent and nondestructive classification was realized for 13 varieties of koi by using the proposed model, and the accuracy rate was 97.9%, which is higher than that of the classical mainstream classification network. This study provides a reference for intelligent classification of marine organisms, and can be extended to the screening and breeding of other species.

**Abstract:**

As the traditional manual classification method has some shortcomings, including high subjectivity, low efficiency, and high misclassification rate, we studied an approach for classifying koi varieties. The main contributions of this study are twofold: (1) a dataset was established for thirteen kinds of koi; (2) a classification problem with high similarity was designed for underwater animals, and a KRS-Net classification network was constructed based on deep learning, which could solve the problem of low accuracy for some varieties that are highly similar. The test experiment of KRS-Net was carried out on the established dataset, and the results were compared with those of five mainstream classification networks (AlexNet, VGG16, GoogLeNet, ResNet101, and DenseNet201). The experimental results showed that the classification test accuracy of KRS-Net reached 97.90% for koi, which is better than those of the comparison networks. The main advantages of the proposed approach include reduced number of parameters and improved accuracy. This study provides an effective approach for the intelligent classification of koi, and it has guiding significance for the classification of other organisms with high similarity among classes. The proposed approach can be applied to some other tasks, such as screening, breeding, and grade sorting.

## 1. Introduction

The diversity of fish resources is an important part of biodiversity and is the basis of the stable and sustainable development of fisheries [1]. As a branch of fish resources, koi variety diversity is conducive to improving the genetic quality of offspring, avoiding inbreeding, and improving the adaptability of koi to the natural environment. To improve the diversity of ornamental fish, classifying koi varieties is a necessary step in breeding koi for biodiversity. The culture of ornamental fish has a long history. As a representative ornamental fish, koi have become increasingly popular since the 1980s. Koi are known as “living gemstones in water”, “geomantic fish”, and “lucky fish” due to their beautiful physique, bright colors, and elegant swimming posture [2]. In the traditional biological breeding process, various genes are selectively gathered to form a new variety through cross-breeding, which can increase species diversity [3]. In the same way, the varieties of koi have gradually evolved into Tancho, Hikariutsurimono, Utsurimono, Bekko, Kawarimono, Taisho Sanshoku, Showa Sanshoku, Asagi, Kohaku, Hikarimoyomono, Koromo, Kinginrin, and Ogon, for a total of thirteen varieties [4,5]. Koi may produce different varieties of offspring through cross-breeding, and each female lays about 300,000 eggs, with an average survival rate of more than 80%. Koi are generally sorted three months after hatching, which is labor-intensive [6]. Additionally, the prices of different varieties of koi greatly vary, so classifying koi is an indispensable step in koi breeding, screening, and grade sorting.

With the improvement in breeding technology and the expansion of breeding scales [7], the market value of koi is increasing. As the price and ornamental value of different koi varieties greatly vary, the screening and grade sorting of koi have become more and more important [8]. However, regardless of variety screening or grade sorting, they both involve classification problems [9,10]. Therefore, many scholars have attached great importance to the research of classification problems for koi varieties. For example, Peng et al. [11] performed the division of Kohaku, Taisho, and Showa varieties, and proposed a four-stage classification method for koi. Song et al. [12] divided three varieties of koi from the perspectives of color, pattern, and lineage. The classification of koi varieties is still manually performed at present, which depends on those with skilled experienced with koi. The traditional manual classification method for koi has disadvantages, such as high subjectivity, low efficiency, and high work intensity. More importantly, classification errors often occur due to the different skills of sorting workers and the subjectivity of classification. Especially when there is high similarity between some varieties of koi, such as Taisho and Showa, misclassification often occurs. To solve the difficulties caused by traditional manual classification, it is necessary to research intelligent classification approaches for koi varieties.

With the development of computer technology, artificial intelligence, and improvements in hardware performance, deep learning technology has rapidly developed [13,14,15]. Recognition technology based on deep learning has especially received attention and been applied in agriculture [16,17], fisheries [18,19,20], medical treatment [21,22,23], and other fields [24,25,26,27]. In the field of fisheries, the existing studies have mainly focused on determining the freshness of marine animals, intelligently recognizing marine organisms, and classifying economic marine organisms. For example, Lu et al. [28] proposed a method of automatically recognizing common tuna and saury based on deep learning, and the final test accuracy of this method was 96.24%. Rauf et al. [29] proposed a fish species recognition framework based on a convolutional neural network, and the classification accuracy of cultured fish (e.g., grass carp, carp, and silver carp) was 92%. Knausgrd et al. [30] proposed a temperate fish detection and classification method based on transfer learning. The classification network was trained on a public dataset for fish (Fish4Knowledge), and the species classification accuracy was 99.27%. From the above research, it is possible to classify koi based on image processing and deep learning technology. The above studies and their related references all discussed methods of classifying underwater animals with high similarity among species.

To the best of our knowledge, there is currently no study on intelligent classification methods for underwater animals with high similarity between varieties. Motivated by the above discussion, we studied a classification approach for underwater animals with high similarity among varieties. The main contributions of this study are as follows: (1) A dataset was created for thirteen kinds of koi. To the best of our knowledge, no koi dataset has yet been reported. (2) A classification problem with high similarity was proposed for underwater animals, and a KRS-Net network was constructed to solve this classification problem for koi with high similarity among varieties. (3) The proposed network could extract deeper koi feature information, and could further improve the classification accuracy by fusing the advantages of support vector machine (SVM) and a fully connected layer. The superiority of this proposed approach was verified through comparison experiments with mainstream networks (AlexNet [31], VGG16 [32], GoogLeNet [33], ResNet101 [34], and DenseNet201 [35]).

## 2. Materials and Methods

### 2.1. Image Acquisition and Data Augmentation

**Figure 1:**
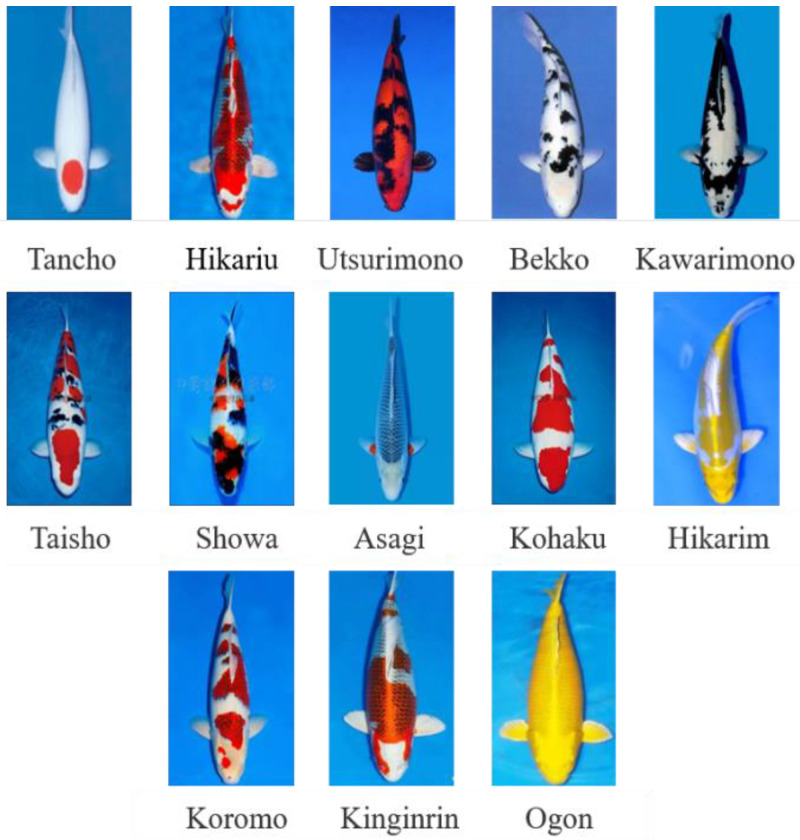
Images of thirteen kinds of koi.

In this study, thirteen kinds of koi were selected as the research objects, and 569 original images of koi with a resolution of 2400 × 1600 were collected using a digital camera (EOS 200D, Canon, Tokyo, Japan). Figure 1 shows representative koi images of the thirteen varieties. For the convenience of drawing and charting, the Hikariutsurimono, Taisho Sanshoku, Showa Sanshoku, and Hikarimoyomono koi varieties are abbreviated as Hikariu, Taisho, Showa, and Hikarim, respectively. The dataset in this study was taken from the actual breeding data of koi, and the method used in this study will also be applied to the actual koi breeding and production.

**Figure 2:**
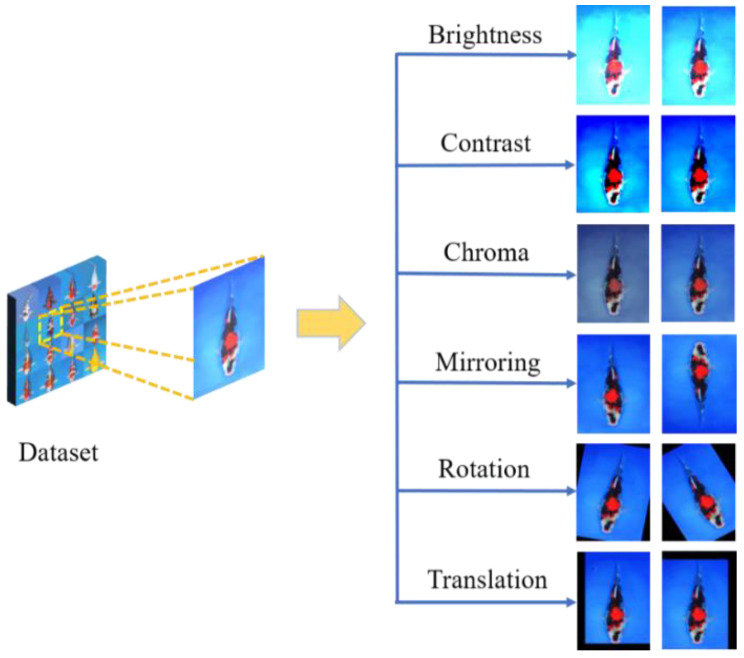
Effect of data augmentation.

To improve the generalization ability of convolutional neural networks, we used image augmentation methods including brightness, contrast, chroma, mirroring, rotation, and horizontal or vertical translation. Generative adversarial network is a kind of efficient augmentation method [36,37]. The images generated by the usual augmentation methods were sufficient to meet the training task of our network. Therefore, the generative adversarial network was not used to expand the dataset in this study. A schematic diagram of the effect of data augmentation is shown in Figure 2.

The dataset had 1464 images after image augmentation, including 1027 images in the training set, 294 images in the verification set, and 143 images in the test set. Because the number of images of Showa and Kohaku was sufficient, we did not perform additional data augmentation processing. The detailed number of images for each variety is shown in Table 1.

### 2.2. KRS-Net Classification Approach

**Figure 3:**
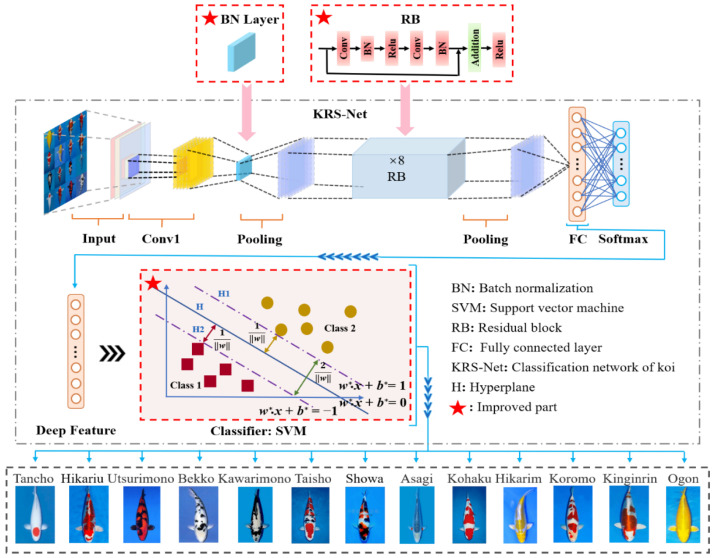
Schematic diagram of the proposed KRS-Net.

Based on the AlexNet framework, a KRS-Net classification network was designed to classify thirteen kinds of koi. KRS-Net is a classification network that is mainly composed of a residual network and SVM. The residual network is used to extract the features of the object, and SVM realizes the classification of objects. A schematic diagram of the proposed network is illustrated in Figure 3.

Based on the AlexNet framework, the main structural changes were as follows:

(1) Replace the original local response normalization (LRN) with batch normalization (BN). Both the LRN layer and BN layer can improve the network generalization ability and training speed, but the performance of the latter is usually superior [37]. Compared with the LRN layer, the BN layer can adapt to a larger learning rate to further improve the network training speed. At the same time, it improves the effect of the regularization strategy, reduces the dependence on the dropout layer, and improves the anti-disturbance ability.

(2) Add eight residual blocks to the network structure. The skip connection in the residual block can overcome the problem of gradient vanishing caused by increasing depth in the network. Therefore, multiple residual blocks were introduced to increase the depth of the network and extract deeper koi feature information. In addition, the difficulty of extracting more subtle koi characteristics is reduced.

(3) Fuse SVM with fully connected (FC) layer to improve accuracy. Inspired by [38], we replaced the softmax layer with SVM to achieve higher generalization model performance, thus improving the accuracy of koi variety classification. To improve the accuracy of classification, the fused SVM with FC was added to the network framework to realize classification of the softmax layer. The fused SVM with FC transforms the nonlinear classification problem into a linear classification problem in high dimensional space by improving the spatial dimensions of the deep feature information extracted from the FC layer. Therefore, the complex processing process of feature information is simplified, and the classification accuracy for koi is further improved.

The information flow of KRS-Net is as follows: First, thirteen kinds of koi images were input into the network after data balance. Second, the koi feature information images was extracted by convolution and pooling, and the extracted feature information was transmitted to the FC layer. The loss rate was reduced by a gradient descent algorithm, and the feature vectors of the FC layer were imported into SVM. Finally, the optimal classification hyperplane was obtained by a kernel function, and the parameters of the network were updated. The process of using the kernel function algorithm to explore the optimal classification hyperplane is as follows:

We assume that the sample space of the training set is $T=\{(x_{i},y_{i}),\;i=1,\;2,\;3,\;\cdots ,\;l\}$ where $x_{i}\in R^{n}$ is the input sample set, $y_{i}\in R^{n}$ is the output sample set, and i is the ith training samples. By adopting the appropriate kernel function $K(x_{i},x_{j})=\varphi (x_{i})\cdot \varphi (x_{j})$ (i=1, 2, ⋯, n ;j=1, 2, ⋯, n ; i≠j), sample x can be mapped into a high dimensional space, where φ(x) represents the dimensional transform function of x. The convex optimization problem with constraints is constructed as:

(1) $$W{\left(\alpha \right)}_{\max}=\sum_{i=1}^{n}\alpha -\frac{1}{2}\sum_{i=1}^{n}\sum_{j=1}^{n}\alpha_{i}\alpha_{j}y_{i}y_{j}K(x_{i},x_{j})$$

(2) $$s.t.\;\sum_{i=1}^{n}\alpha_{i}y_{i}=0\;(i=1,2,3,\cdots ,n),\;0\leq \alpha_{i}\leq C$$

where $W{\left(\alpha \right)}_{\max}$ is the optimized object function; C is the penalty parameter; α is the Lagrange multiplier, and its optimal solution is $\alpha^{*}=(\alpha_{1}^{*},\alpha_{2}^{*},\cdots ,\alpha_{n}^{*}{)}^{T}$, which can be obtained by Formulas (1) and (2). Furthermore, we construct the hyperplane for classification. The optimal classification hyperplane is defined as follows:

(3) $$w^{*}\cdot x+b^{*}=0$$

(4) $$w^{*}=\sum_{i=1}^{n}\alpha_{i}^{*}y_{i}x_{i}$$

(5) $$b^{*}=y_{j}-\sum_{i=1}^{n}\alpha_{i}^{*}y_{i}K(x_{i},x_{j}),\;s.t.\;0\leq \alpha_{j}^{*}\leq C$$

where $w^{*}$ is the normal vector to the optimal hyperplane, and $b^{*}$ is the offset.

Then, the categories of koi varieties can be determined through the classification decision function, which is defined as:

(6) $$f\left(x\right)=\mathrm{sign}\left[\sum_{i=1}^{n}\alpha_{i}^{*}y_{i}K\left(x_{i},x_{j}\right)+b^{*}\right]$$

## 3. Experimental Results and Analysis

### 3.1. Setup of Experiment and Performance Indexes

The training work of KRS-Net proposed in this paper was implemented in MATLAB2020a (MathWorks, Natick, MA, USA). The computer performance parameters for network training were as follows: CPU: Intel (R) Xeon (R) E5-4627v4@2.60 GHz; GPU: NVIDIA RTX 2080Ti; RAM: 64 G; OS: Windows10 (Lenovo, Hong Kong, China).

To unify the training environment and avoid interference from other factors, the experiments were conducted under the same conditions.

KRS-Net is trained on the established dataset of koi. The learning rate of network training was uniformly set to 0.0001 according to [39]. Because the values of batch size and epoch affect the training effect to a certain extent, we studied the influence of the batch size and epoch on the effect of the network training under different hyperparameters and obtained the maximum classification performance of the network. The experimental results are shown in Table 2.

**Figure 4:**
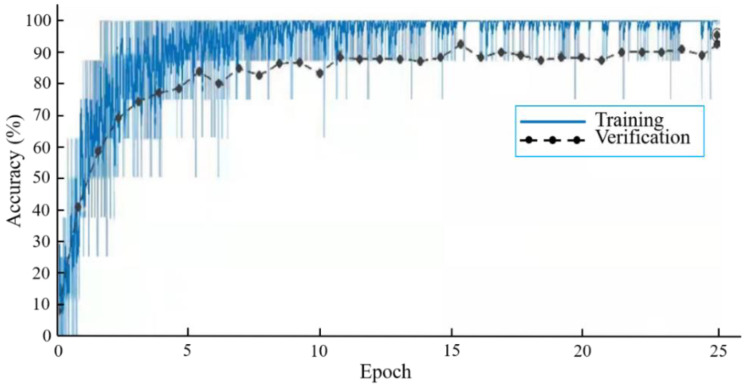
Accuracy curves.

**Figure 5:**
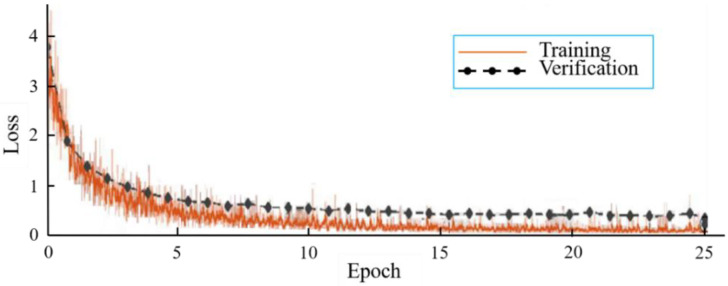
Loss curves.

From Table 2, the best classification test accuracy of KRS-Net was 97.90% when the batch size was set to 8 and the epoch was set to 25. When the batch size was 8 and the epoch is 25, Figure 4 shows the training and verification accuracy curves of KRS-Net. From Figure 4, we can see that the verification curve is close to the training curve, which indicates that the network had better performance. The loss curves of KRS-Net in the training and verification processes are shown in Figure 5.

**Figure 6:**
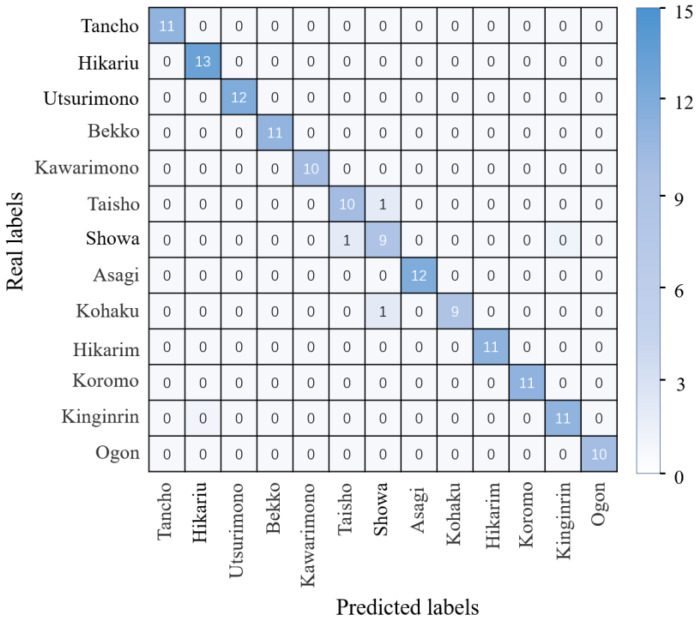
Confusion matrix.

To further show the specific koi classification with the proposed approach, 143 images were used to test the trained KRS-Net. The real and predicted koi categories are summarized in the form of a matrix in Figure 6. Figure 6 shows that each value is the largest in the diagonal line in the same columns, which indicates that the KRS-Net had a better classification effect.

### 3.2. Visualization of Features

We visualized the features to make the extracted features more intuitive from three aspects: different network layers, single image, and gradient-weighted class activation mapping (Grad-CAM).

**Figure 7:**
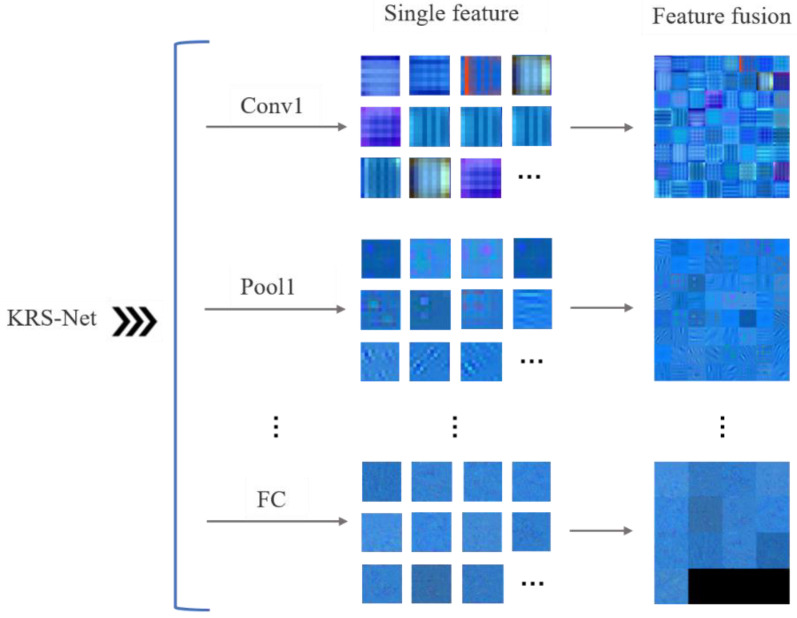
Feature visualization of KRS-Net.

To reflect the features learned by the convolutional neural network in the training process, the single and fusion features were extracted and visualized for different network layers of KRS-Net, as shown in Figure 7.

In Figure 7, the single feature and fusion feature are visualized for the first convolution layer (Conv1), first pooling layer (Pool1), and the FC layer of KRS-Net from the shallow to the deep layer. The fused feature is a new combined feature created on the basis of multiple single features, and the fused features reflect the correlation information among the single features. Eliminating the redundant information caused by the correlation between single features is helpful for the subsequent classification decision making of the network. From Figure 7, we can see that some low-level image feature information was extracted by the convolution layer and pooling layer at the front of the network, such as koi color; and some high-level image feature information was extracted by the FC layer at the end of the network.

**Figure 8:**
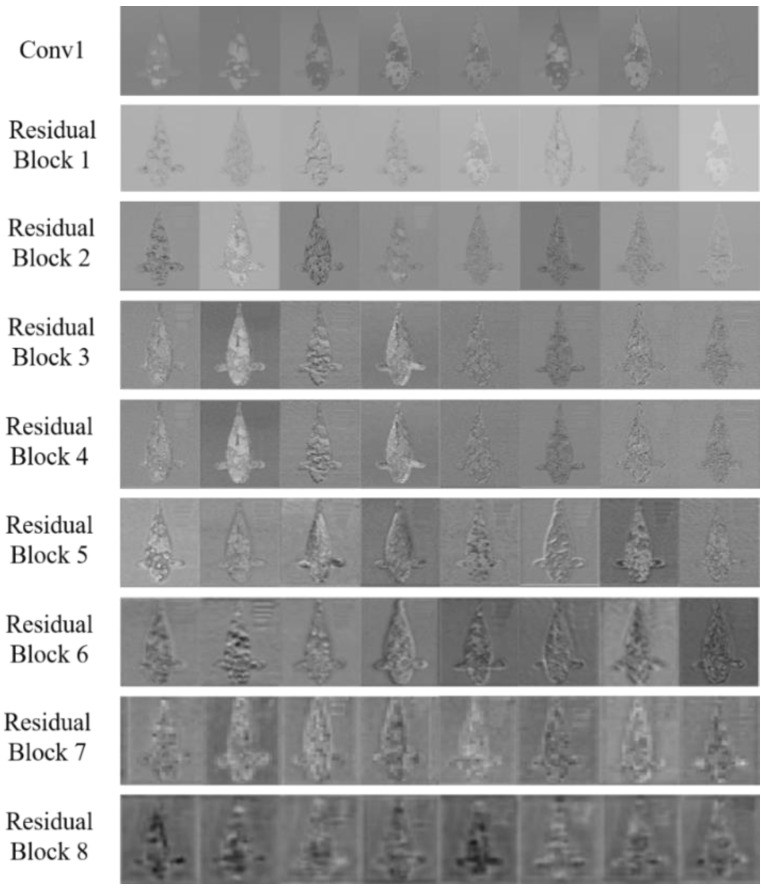
Activation regions of KRS-Net in a single image.

Different activation regions are generated at each layer due to the differing abilities to extract image features at each network layer when the image is input into the convolutional neural network. Furthermore, the features learned by each layer can be intuitively seen by comparing the activation regions with the original image. To study the intermediate processing process of KRS-Net for a single image, the activation regions are successively shown for the first convolution layer and eight residual blocks by considering a Showa example in Figure 8.

From Figure 8, it can be seen that the shallow network extracted the simple features of images, and the extracted features became more complex and abstract with the increase in network depth.

**Figure 9:**
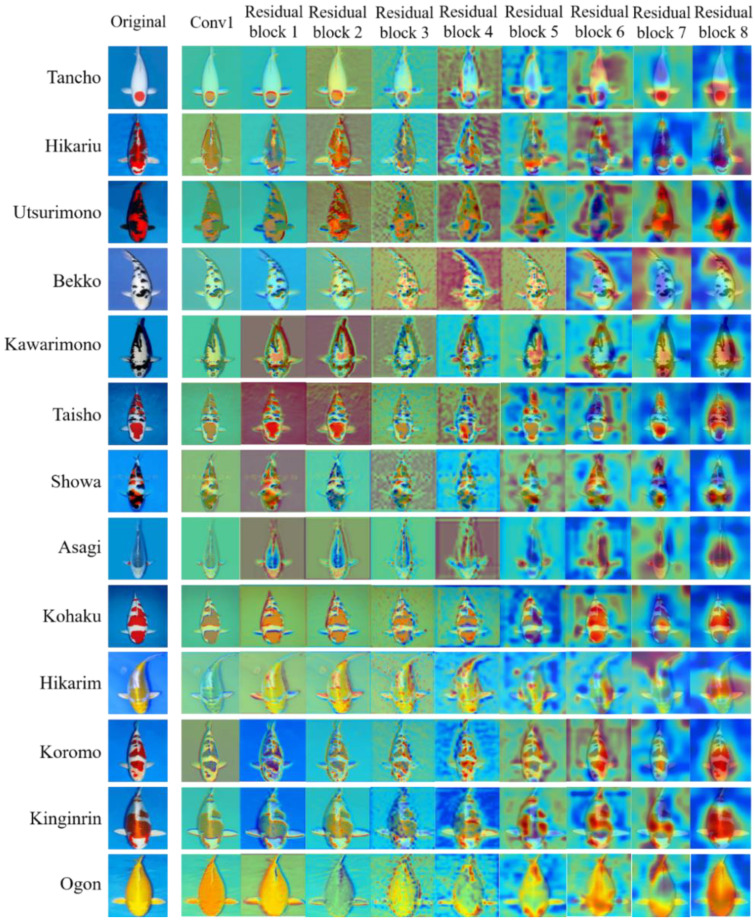
Grad-CAM visualization of KRS-Net.

Grad-CAM [40] can visualize a region of interest of an image, which helped to understand how the convolutional neural network makes decisions on the classification of koi. Figure 9 gives the Grad-CAM visualization of the first convolution layer and eight residual blocks in KRS-Net for koi. In Figure 9, the red region of Grad-CAM provides an important basis to make classification decisions on the input image for the network, and the blue region is the second part. With the increase in network depth, the red region gradually focuses on the special characteristics of the object. Taking the image of Tancho as an example in Figure 9, we can see that the red region of the output image of residual block 4 is relatively scattered, but the red region slowly focuses on the round spot on the head of Tancho with the deepening of the network, which is the most obvious feature that distinguishes this variety from other varieties. As can be seen from Figure 9, the network could effectively capture the characteristics of each koi variety, so that the classification task was completed well.

### 3.3. Comparative Analysis with Other Classification Networks

**Figure 10:**
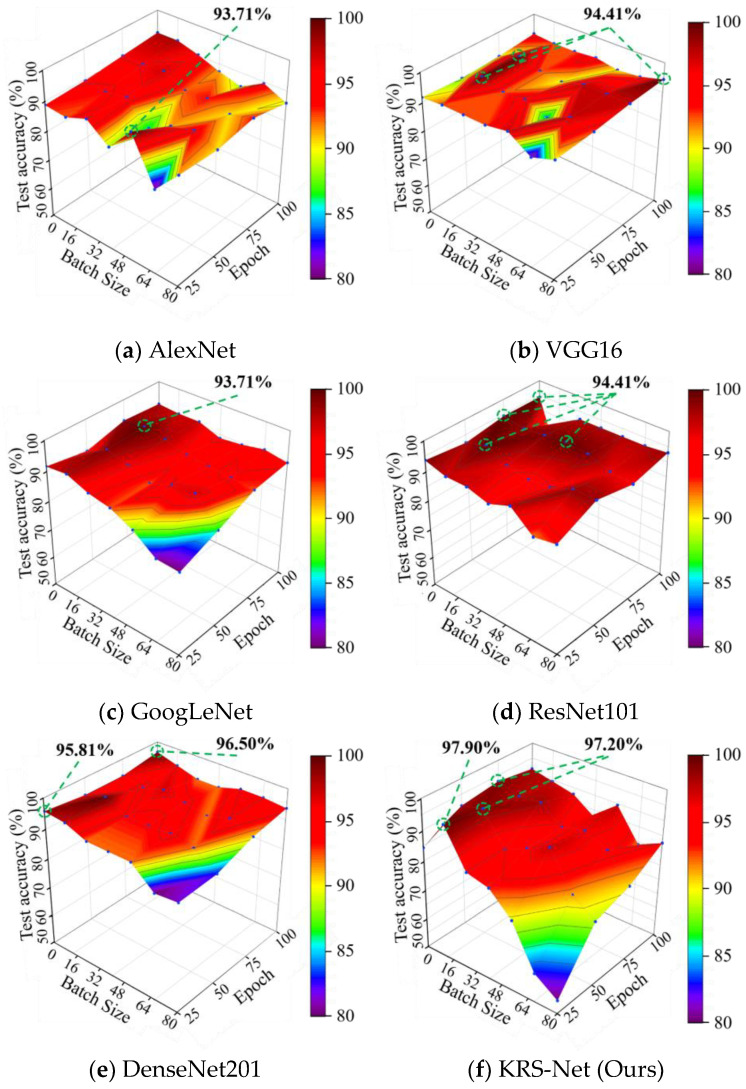
Test accuracies of different networks.

To verify the superiority of the proposed approach, the test accuracy of the KRS-Net was compared with that of some mainstream classification networks such as AlexNet, VGG16, GoogLeNet, ResNet101, and DenseNet201. To visually display the comparison results, a 3D colormap surface was used to study the influence of hyperparameters on the test accuracy of the networks. Figure 10 shows the 3D colormap surfaces of the test accuracy of KRS-Net and that of the other five classification networks.

As can be seen from Figure 10, the highest test accuracy of AlexNet, VGG16, GoogLeNet, ResNet101, DenseNet201, and the proposed KRS-Net was 94.41%, 94.41%, 93.71%, 93.71%, 96.50%, and 97.90%, respectively. The results of the comparative analysis showed that the test accuracy of KRS-Net was 1.4% higher than the highest test accuracy of the other five classification networks, which proves the superiority of the proposed approach. Notably, the classification effect of the six networks gradually increased with the decrease in batch size. For this phenomenon, a specific analysis is provided in Section 4.

The accuracy, precision, recall, and *F*1 were selected as performance evaluation indexes to further analyze the koi classification performance of the networks, whose definitions are as follows [41]:

(7) $$Accuracy=\frac{TP+TN}{TP+TN+FP+FN}$$

(8) $$Precision=\frac{TP}{TP+FP}$$

(9) $$Recall=\frac{TP}{TP+FN}$$

(10) $$F1=\frac{2\times Pr\times Re}{Pr+Re}$$

where *TP*, *TN*, *FP*, and *FN*, respectively, represent true positive, true negative, false positive, and false negative. Accuracy is an intuitive evaluation index, representing the proportion of the number of koi samples correctly classified to the total number of koi samples. Precision represents the proportion of the number of real positive samples to the number of positive samples predicted by the network. Recall represents the proportion of positive samples predicted by the network to all positive samples. *F*1 is a comprehensive evaluation index based on precision and recall. A larger *F*1 value indicates better network performance.

The comparative experimental results are shown in Table 3. The proposed KRS-Net had a better effect when the batch size was eight, as shown in Table 2. So, the batch size was set to 8 and the epoch was set to 25, 50, 75, or 100 in the experiment. The following performance evaluation indexes of each network represent the average values of thirteen koi varieties, but not the performance evaluation index of a single variety.

It can be seen from Table 3 that when the batch size of KRS-Net was set to 8 and the epoch was set to 25, the classification accuracy, precision, recall, and *F*1 were 99.68%, 97.90%, 97.76%, and 97.80%, respectively, which are all higher than those of the other five classification networks. In addition, we can see that the four evaluation indices of the network all decreased with the increase in epochs, as shown in Table 3, which may have occurred because the network gradually generated overfitting with the increase in epoch number in the subsequent training process.

Remark: there is a kind of fine-grained image classification method (subcategory image classification method), which we used to divide coarse-grained categories into more detailed subclasses according to the differences in some special parts among subclasses. However, the difference between the subclasses of koi lies not only in some special parts, but also in the shape and position of its body patterns as well as the ratio of red, white, and black. Considering the above factors, we did not choose a similar algorithm such as a fine-grained algorithm.

## 4. Discussion

### 4.1. Factors Influencing Test Accuracy

Although the test accuracy of the proposed KRS-Net reached 97.90%, there were still some koi varieties that were misclassified. The reasons for the misclassification may include the following several aspects: (1) Some images were not clear enough because koi swim quickly. Additionally, the part of the image containing the typical characteristics may have been incomplete due to the differences in shooting angle during the data acquisition, which may have affected the training effect of the network. (2) The water ripples generated by the swimming of koi may have affected the image definition, which resulted in the blurring and distortion of images, as well as other problems [42]. (3) The cross-breeding of koi reduces the differences between varieties, resulting in a situation where the offspring are neither like the characteristics of their mother fish nor the various characteristics of their father, which poses difficulties for classification.

### 4.2. Influence of Batch Size on Classification Performance

From the 3D colormap surface in Figure 10, we can see that the batch size in the convolutional neural network had a greater influence on the classification performance of the network, which was also obtained in [43]. The test accuracies of six classification networks all decreased with the increase in batch size. A similar phenomenon was shown in [44]. This phenomenon may be caused by the larger batch size of training data being not conducive to parameter updating and optimization. On the contrary, a smaller batch size may be better for solving the positive and negative cancellation problem of the gradient update value caused by the differences in sampling.

### 4.3. Advantages of KRS-Net in Structure

The test accuracy of the proposed KRS-Net was higher than that of AlexNet, VGG16, GoogLeNet, ResNet101, and DenseNet201 in this study, which was determined by the advantages of the KRS-Net structure. First, the original LRN is replaced by BN based on AlexNet architecture, which reduces the complexity of the network and improves the convergence of the network. Second, the addition of residual block deepens the network, which can extract deeper information and effectively overcome the gradient vanishing problem. Third, the fusion of SVM with a FC layer replaces the softmax classifier in AlexNet, which transforms the original nonlinear classification problem into a linear classification problem to deal with high-dimensional space so that the test accuracy is further improved.

### 4.4. Influence of Structure on Training Time and Parameters

To identify the factors affecting the network training time and parameter quantity, we studied the training time and parameters of the six networks when the batch size was 8 and the epoch is 25, as shown in Table 4. It can be seen from Table 4 that there was a high similarity in training time between KRS-Net and the lightweight network AlexNet, but the test accuracy of the former was 4.19% higher than that of the latter. This may be because the addition of the BN layer improves the training speed and convergence of the network. In addition, KRS-Net has more network layers and connections but has a smaller network size and fewer parameters than VGG16 and AlexNet. This may be because the skip connection structure of the residual network not only overcomes the gradient vanishing problem but also reduces the network size and parameters.

### 4.5. Future Work

The evolution of koi has become more and more complex through many years of breeding and screening. To date, koi can be divided into thirteen categories in a narrow sense, but more than 100 subcategories have broadly been bred. If more than 100 varieties of koi can effectively be classified, the time cost and labor force required for koi breeding will be further reduced to a certain extent. Therefore, a multi-variety and lightweight classification network will be studied with a high accuracy rate and rapid speed in future work to lay the foundation for the research and development of multi-variety classification equipment.

The actual situation for the classification of koi varieties may be complex. Therefore, a multi-objective situation may occur, and some factors (such as posture change of koi, object occlusion, and illumination change) affect the classification accuracy of koi varieties. Our future work will focus on solving the problems of classifying koi varieties in complex situations.

## 5. Conclusions

Koi variety classification was studied to solve the problems caused by the high similarity among some varieties. In this study, a dataset including thirteen kinds of koi was established, and a koi variety classification network KRS-Net was proposed based on residual network and SVM. Compared with five other mainstream networks, the performance superiority of the proposed KRS-Net was proven. this study provides a new solution for the classification of koi varieties, which can be extended to breeding, aquaculture, grade sorting, and other marine fields.

## Figures and Tables

**Table 1:** Numbers of images of thirteen koi varieties.

Koi Variety	Original Images	Augmented Images	Images after Augmentation
Tancho	37	74	111
Hikariu	42	84	126
Utsurimono	25	100	125
Bekko	22	88	110
Kawarimono	21	84	105
Taisho	75	39	114
Showa	104	0	104
Asagi	25	100	125
Kohaku	101	0	101
Hikarim	22	88	110
Koromo	23	92	115
Kinginrin	49	64	113
Ogon	21	84	105
Total	567	897	1464

**Table 2:** Experimental test results of KRS-Net.

Hyperparameter	Epoch = 25	Epoch = 50	Epoch = 75	Epoch = 100
Batch Size = 4	93.71%	96.50%	97.20%	96.50%
Batch Size = 8	**97.90%**	97.20%	96.24%	95.80%
Batch Size = 16	93.01%	93.01%	96.50%	95.10%
Batch Size = 32	92.31%	94.41%	94.41%	93.01%
Batch Size = 64	83.92%	90.91%	93.71%	91.61%

**Table 3:** Performance evaluation of networks.

Evaluation Index (%)	AlexNet	VGG16	GoogLeNet	ResNet101	DenseNet201	KRS-Net
**Epoch = 25**						
Accuracy	98.17	98.82	98.82	98.71	99.25	**99.68**
Precision	90.51	92.37	92.72	92.98	95.46	**97.90**
Recall	87.55	91.96	91.89	91.19	94.83	**97.76**
*F*1	86.97	91.58	91.00	90.53	94.78	**97.80**
**Epoch = 50**						
Accuracy	97.63	99.19	98.92	99.14	99.57	99.57
Precision	90.69	94.97	93.41	95.30	97.80	97.58
Recall	88.25	94.69	92.59	93.99	96.92	97.13
*F*1	87.10	94.29	91.79	93.47	96.87	97.12
**Epoch = 75**						
Accuracy	98.60	98.87	98.39	98.92	98.92	99.35
Precision	91.98	95.05	94.24	94.54	94.68	96.49
Recall	90.49	93.91	93.43	92.52	92.52	95.84
*F*1	89.93	92.87	93.01	91.67	91.88	95.90
**Epoch = 100**						
Accuracy	98.81	98.82	98.71	95.91	99.16	99.41
Precision	93.51	95.07	92.24	88.00	96.03	96.40
Recall	91.89	90.71	91.26	72.38	93.92	96.54
*F*1	91.57	91.36	90.82	74.06	93.23	96.17

**Table 4:** Parameters and training times of six classification networks.

Networks	Training Time (s)	Number of Layers	Number of Connections	Size of Network (MB)	Parameters (M)
AlexNet	1079	25	24	227.00	61.00
VGG16	1112	41	40	515.00	138.00
GoogLeNet	1403	144	170	27.00	7.00
ResNet101	3320	347	379	167.00	44.60
DenseNet201	8864	708	805	77.00	20.00
**KRS-Net**	**1338**	**71**	**78**	**49.70**	**10.89**

## Data Availability

With the consent of the authors, the data to this article can be found online at: http://33596lk211.qicp.vip/dataweb/index.html, accessed on 21 May 2022.
